# Induced Foxp3^+^ T Cells Colonizing Tolerated Allografts Exhibit the Hypomethylation Pattern Typical of Mature Regulatory T Cells

**DOI:** 10.3389/fimmu.2016.00124

**Published:** 2016-04-11

**Authors:** Robert Hilbrands, Ye Chen, Adrian R. Kendal, Elizabeth Adams, Stephen P. Cobbold, Herman Waldmann, Duncan Howie

**Affiliations:** ^1^Therapeutic Immunology Group, Sir William Dunn School of Pathology, University of Oxford, Oxford, UK

**Keywords:** Foxp3, regulatory T cells, hypomethylation, transplantation, tolerance

## Abstract

Regulatory T cells expressing the transcription factor Foxp3 require acquisition of a specific hypomethylation pattern to ensure optimal functional commitment, limited lineage plasticity, and long-term maintenance of tolerance. A better understanding of the molecular mechanisms involved in the generation of these epigenetic changes *in vivo* will contribute to the clinical exploitation of Foxp3^+^ Treg. Here, we show that both *in vitro* and *in vivo* generated antigen-specific Foxp3^+^ Treg can acquire Treg-specific epigenetic characteristics and prevent skin graft rejection in an animal model.

## Introduction

Regulatory T cells expressing the transcription factor Foxp3 (Foxp3^+^ Treg) develop in both the thymus (tTreg) and the periphery (pTreg). Together, they form a pool of naturally arising Treg (nTreg) that form an essential part of the immune system ensuring adequate immune homeostasis *via* control of inflammation and diverse immune responses (1–4). Induction, or restoration, of immune tolerance through harnessing of Foxp3^+^ Treg has been achieved in many animal models. However, translation toward the clinic has been limited, likely due to a lack of understanding of the molecular mechanisms required for Treg generation and long-term maintenance. Recently, Ohkura et al. showed that two independent processes are required for the development of stable Foxp3^+^ Treg. These are the induction of Foxp3 expression, and the installation of a Treg-specific hypomethylation pattern to establish lineage commitment (5). Only those T-cells demonstrating both processes are committed to the Treg cell lineage and to stable function. Better understanding of how these processes occur would enable generation, both *in vitro* and *in vivo*, of Foxp3^+^ Treg that are functionally stable, an essential requirement for their therapeutic application (6, 7).

*In vitro* generation of Foxp3^+^ Treg (iTreg) requires TCR stimulation of naive CD4^+^ T cells in the presence of TGF-β, but is in itself insufficient to establish those Treg-specific epigenetic changes. As a consequence, iTreg appear unstable with rapid loss of Foxp3 expression when transferred *in vivo*, accompanied by the production of diverse proinflammatory cytokines. This is in sharp contrast to the apparent stability of Foxp3 expression of naturally occurring mature Treg (5, 8, 9). Inhibition of DNA methylation within iTreg using azacytidine stabilizes Foxp3 expression and enhances demethylation of the Treg-specific demethylated region (TSDR) (10). Together, these data have demonstrated a significant contribution of the Treg-specific hypomethylation pattern to lineage stability. Although administration of IL2/anti-IL2 complexes helps to stabilize Foxp3 expression in iTreg *in vivo* with increased demethylation of the TSDR, it remains incompletely understood how iTreg can be stabilized and acquire epigenetic maturation *in vivo* (11).

Harnessing TGF-β-dependent pTreg within the body to achieve antigen-specific tolerance is now an attractive therapeutic goal. We have previously demonstrated that TCR-transgenic RAG^−/−^ mice, lacking Treg, can still be tolerized to skin grafts carrying the nominal antigen. This was achieved through coreceptor blockade with anti-CD4 antibodies (12, 13). Transplantation tolerance in this model absolutely depended on the induction of Foxp3^+^ pTreg and their sustained presence (14). The induction of pTreg in this system also depends on the presence of active TGF-β (13).

Here, we use this TCR-transgenic mouse model to determine the requirements for both sustained Foxp3 expression and Treg-specific hypomethylation in the setting of acquired transplantation tolerance. We investigated these requirements both for *in vitro* generated antigen-specific iTreg after adoptive transfer in lymphopenic hosts bearing skin grafts and for *in vivo* generated antigen-specific pTreg following anti-CD4 blockade. Together, our data demonstrate that renewed antigen stimulation by the tolerated tissue enhances the number of T-cells that demonstrate a stable Treg-specific hypomethylation pattern.

## Materials and Methods

### Experimental Mice

B6.Foxp3^hCD2^ knock-in, RAG1^−/−^ Marilyn.Foxp3^hCD2^ knock-in, B6.RAG1^−/−^, and CBA.RAG1^−/−^ were bred and maintained under specific pathogen-free conditions in the animal facility of the Sir William Dunn School of Pathology. B6.Foxp3^hCD2^ knock-in and RAG1^−/−^ Marilyn.Foxp3^hCD2^ knock-in were generated, as described previously (14).

### Transplantation and Adoptive Cell Transfer

All animal procedures were performed in accordance with the Home Office Animals (Scientific Procedures) Act of 1986 under project license numbers PPL 30/2549 and PPL 30/3060. Local ethical committee approval was also obtained. Skin grafting was conducted with full thickness skin tail (1 cm × 1 cm) on the lateral flank, as previously described (15). Grafts were observed daily after cast was removed at day 8 and considered rejected when no viable skin was present. Adoptive transfer of sorted or unsorted cells was achieved by tail vein injection using an appropriate volume of washed cell suspension in sterile 1× PBS. In some experiments, CBA.RAG^−/−^ mice were used as donors to C57.Bl/6 recipients, so that the color difference left no doubt about rejection *via* the indirect pathway.

### Bone Marrow Dendritic Cell Preparation

C57Bl/6 bone marrow was sieved through a 70-μm nylon mesh in R10 medium (RPMI 1640, 10% FCS, 2 mM l-glutamine, 50 U/ml penicillin, 50 μg/ml streptomycin, 5 × 10^−5^M, and 50 μM 2-mercapto-ethanol), and cells plated at 7.5 × 10^6^/10 cm plate (Corning Inc., Corning, NY, USA), supplemented with ~25 ng/ml murine recombinant GM-CSF, supplied as culture supernatant. Medium was replaced on days 3 and 6. Bone marrow dendritic cells (BMDCs) were replated on day 6 and harvested on day 7 by gentle pipetting.

### *In Vitro* Treg Generation

RAG1^−/−^ Marilyn.Foxp3^hCD2^ spleen cells were harvested and prepared, including red blood cell lysis, under sterile conditions. About 5 × 10^5^ RAG1^−/−^ Marilyn.Foxp3^hCD2^ cells were cultured at 37°C and 6% of CO_2_ in 2 ml of R10 medium [RPMI 1640 medium (Lonza) + 10% vol/vol FCS (Invitrogen) + 50 μg/ml penicillin + streptomycin (Invitrogen) + 2 mM l-glutamine (Invitrogen) + 50 μM 2-mercapto-ethanol], 2 ng/ml TGF-β, 1 × 10^5^ B6.WT BMDCs, and 100 nM of male H-Y peptide (Dby H-2Ab; NAGFNSNRANSSRSS). Cultures were harvested at day 7.

### *In Vivo* pTreg Generation

Female RAG1^−/−^ Marilyn.Foxp3^hCD2^ mice were grafted with male B6.RAG1^−/−^ skin and treated with three doses of 1 mg non-depleting anti-CD4 (YTS 177.9) over 7 days. Mice were sacrificed at week 10. Spleen, non-draining and draining lymph nodes were harvested separately and prepared for flow cytometry. Subsequently, samples were pooled to obtain sufficient cell numbers for MoFlo sorting and DNA methylation analysis.

### Flow Cytometry Staining and Cell Isolation

Spleen, draining and non-draining lymph nodes were labeled directly with fluorescently conjugated antibodies. Four-color analysis was performed using a FACS Calibur (BD) with dual laser (488 and 633 nm) excitation. The analysis gate was set on the forward and side scatters to eliminate cell debris and dead cells. For cell isolation, cells were purified from pooled spleens and lymph nodes using a 70-μm cell strainer. Cells were stained using mouse monoclonal anti-hCD2 PE conjugated antibodies RPA-2.10 (BD Pharmingen), rat anti-CD4 APC conjugated antibodies RMA4–5 (BD Pharmingen), and hamster anti-TCR PerCP conjugated H57-597 (BD Pharmingen) conjugated antibodies. Single stain samples were also prepared for MoFlo calibration before cell sorting. Cells were MoFlo cell sorted as per manufacturer’s guidelines.

### DNA Methylation Analysis

Bisulfite converted DNA was obtained from MoFlo sorted cells using Epitect Fast LyseAll Bisulfite conversion kit (Qiagen). Converted DNA was amplified by PCR and subcloned with TOPO^®^TA cloning kit (Invitrogen). PCR primers for TSDR were designed using MethPrimer software, Forward primer: TTTTGGGTTTTTTTGGTATTTAAGA, and Reverse primer: AACCAACCAACTTCCTACACTATCTAT. Primers for *Tnfrsf18* exon 5, *Ctla4* exon 2, and *Eos (Ikzf4)* intron 1b were used as described by Ohkura et al. Clones (*n* = 10–30) were incubated overnight in 2 ml LB medium with ampicillin and screened for the presence of insert using PCR. When the insert was present, plasmids were extracted using Miniprep Kits (Qiagen) and sequenced. Visualization and quality control for DNA methylation of sequenced clones were performed with BiQ Analyzer [(16), p. 4067–8].

## Results

### iTreg Can Acquire Epigenetic Maturity *In Vivo*

Based on a genome-wide DNA methylation analysis, Ohkura et al. (5) established that the identity of naturally occurring Treg (nTreg, which comprise both tTreg and pTreg) is characterized by a Treg-specific hypomethylation pattern on four genomic regions located in genes encoding for Treg-specific proteins. These include the *Foxp3* TSDR (TSDR–FOXP3), *Tnfrsf18* exon 5 (GITR), *Ctla4* exon 2 (CTLA-4), and *Eos (Ikzf4)* intron 1b (EOS). Together, they are defined as “nTreg-Me” (5).

**Figure 1:**
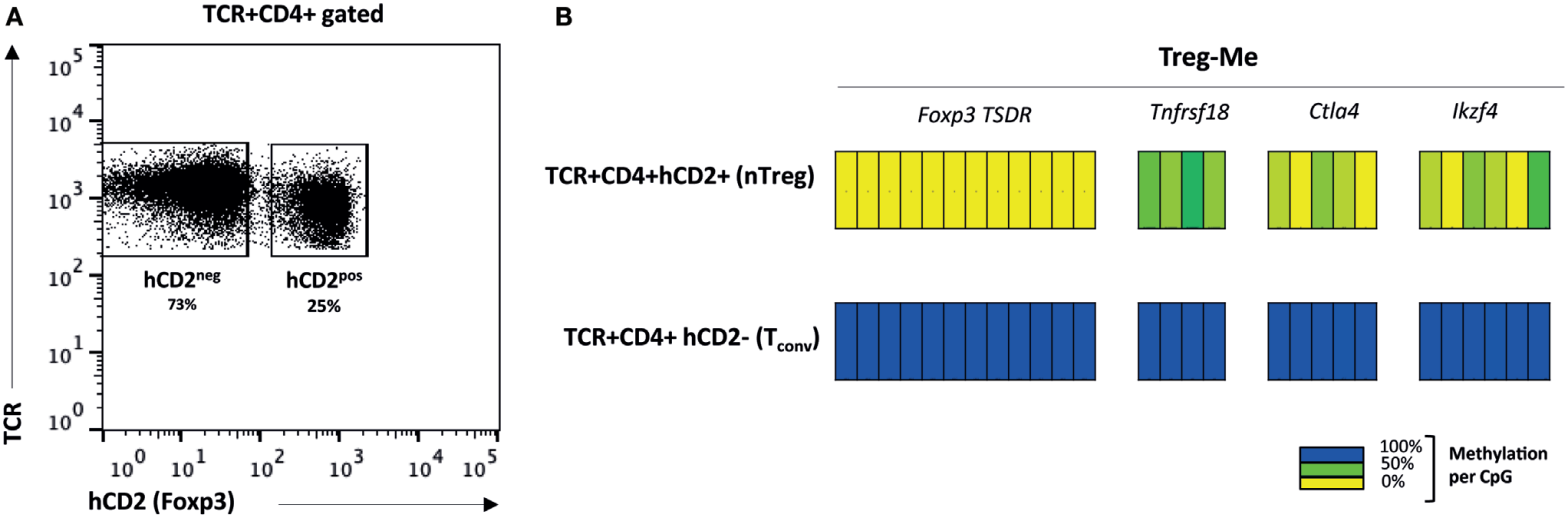
**nTreg are characterized by a specific hypomethylation pattern**. **(A)** Naturally occurring TCR^+^CD4^+^Foxp3^+^ Treg (nTreg) and TCR^+^CD4^+^Foxp3^−^ conventional T cells (Tconv) were isolated from spleen and lymph nodes of B6.Foxp3^hCD2^ knock-in mice. **(B)** CpG methylation status of established Treg-specific gene regions (nTreg-Me). TSDR, Treg-specific demethylated region. Data representative of five separate experiments.

First, we studied this Treg-specific hypomethylation pattern in nTreg obtained from B6.Foxp3^hCD2^ reporter mice (14). These mice have an identical genetic background to wild-type C57BL/6 (B6) mice and allow reliable isolation of live Foxp3^−^ and Foxp3^+^ populations for cell sorting and adoptive transfer studies (14). We isolated conventional CD4^+^ T cells (TCR^+^CD4^+^hCD2[Foxp3]^−^) and nTreg (TCR^+^CD4^+^hCD2[Foxp3]^+^) from spleen and lymph nodes (Figure 1A). Bisulfite sequencing of selected DNA regions demonstrated hypomethylation at nTreg-Me loci, whereas these regions remained completely methylated in conventional (Foxp3^−^) CD4^+^ T cells (Figure 1B). This confirms the earlier findings of Ohkura et al. (5) and further illustrates that nTreg derived from the B6.Foxp3^hCD2^ reporter mice are an epigenetically homogenous population of Foxp3^+^ Treg. In female mice, the methylation status of the TSDR was around the expected 50% based on the non-functional Foxp3 gene on the (inactivated) X-chromosome (Figure S1 in Supplementary Material). This “quantitation” allowed us to set the baseline expected for any demethylated Foxp3^+^ T-cells developing in female TCR-transgenic mice.

**Figure 2:**
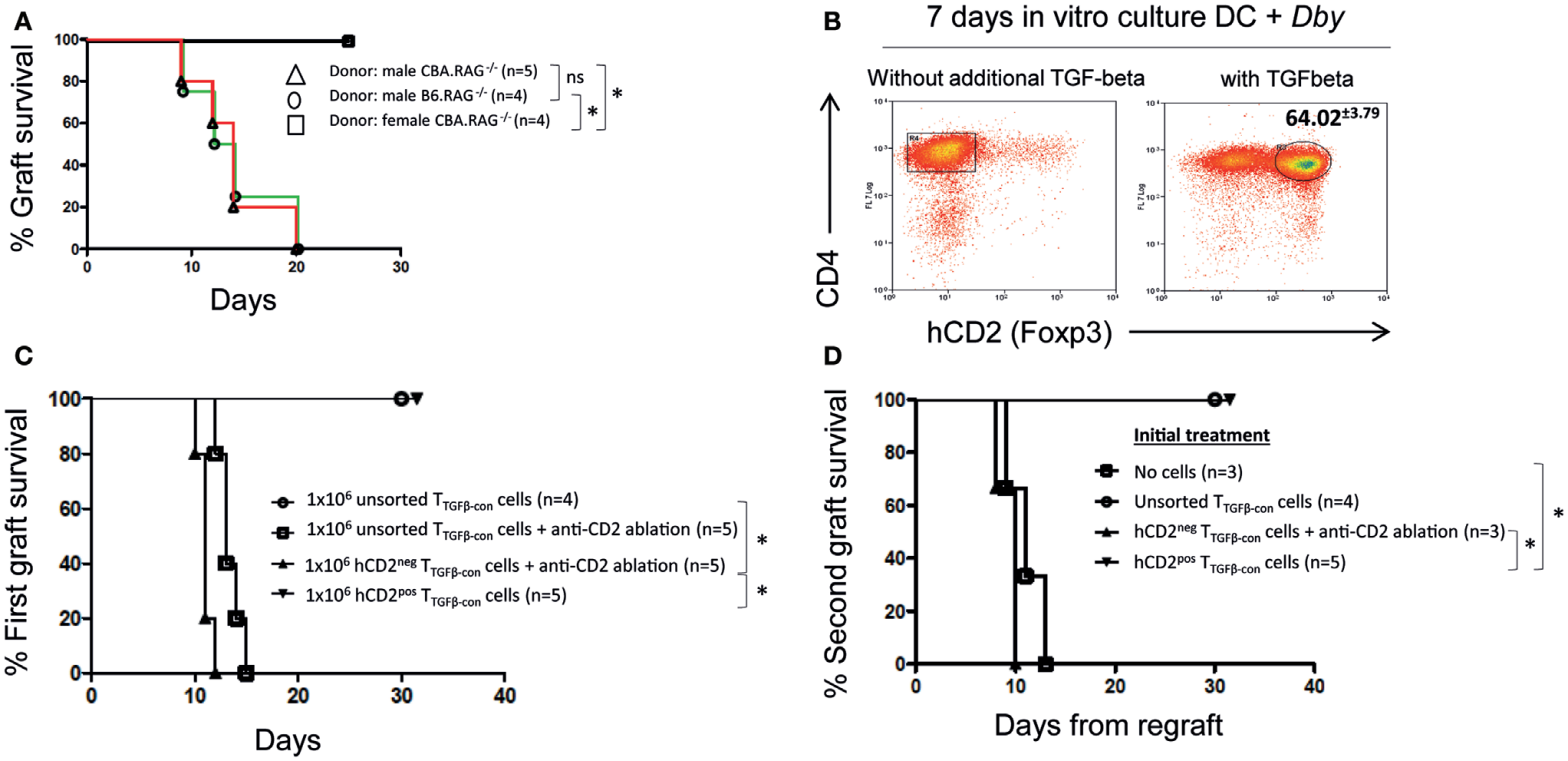
**Antigen-specific TGF-β conditioned iTreg prevent graft rejection**. **(A)** B6.RAG1^−/−^ Marilyn.Foxp3^hCD2^ knock-in mice reject skin grafts from male B6.RAG1^−/−^ and CBA.RAG1^−/−^ mice. Female CBA.RAG1^−/−^ lack male antigen and are accepted. Statistical analysis Log-Rank test; *p < 0.05, ns, not significant. **(B)** Generation of Dby-specific *in vitro*-induced Foxp3^+^ Treg (iTreg) from splenocytes derived from female B6.RAG1^−/−^ Marilyn.Foxp3^hCD2^ mice in the presence or absence of additional TGF-β (2 ng/ml), Dby peptide, and female B6 wild-type derived bone marrow dendritic cells. Data representative of at least three experiments. **(C)** 1 × 10^6^ unsorted and sorted TGF-β conditioned cells (iTreg) were adoptively transferred to lymphopenic B6.RAG1^−/−^ mice with or without hCD2 ablation treatment. Mice received skin grafts from male CBA.RAG1^−/−^ mice. Statistical analysis Log-Rank test; *p < 0.01. **(D)** Mice received an initial male CBA RAG1^−/−^ skin graft and treatment as indicated. Thirty days following the first skin graft, 10^5^ Marilyn CD4 T cells were injected i.v. and a fresh male CBA RAG^−/−^ skin graft was transplanted on the contralateral flank of recipients. Statistical analysis Log-Rank test; *p < 0.01. All data representative of three separate experiments.

Next, we investigated the stability of Foxp3 expression and epigenetic characteristics of antigen-specific iTreg derived from naive CD4^+^ T cells cultured with antigen and TGF-β *in vitro*. We made use of female TCR-transgenic C57BL/6 Rag^−/−^ Marilyn.Foxp3^hCD2^ mice (14) that exhibit only CD4^+^ lymphocytes with a single TCR specificity recognizing the male *Dby* peptide. These mice have no nTreg in their thymus or periphery. They reject skin grafts bearing the male antigen expressed on both a B6 Rag1^−/−^ and CBA Rag1^−/−^ background (the latter through indirect presentation only and used for easy scoring of rejection), but do not reject grafts from female donors (Figure 2A). As few as 2500 Marilyn CD4^+^ naive T cells adoptively transferred into a female B6 Rag1^−/−^ recipient gives a prompt rejection of all male skin grafts by day 10.

Marilyn.Foxp3^hCD2^ T-cells provide a useful source of antigen-specific iTreg developing after an *in vitro* stimulation with DCs and antigen in the presence of TGF-β (Figure 2B). Both unsorted TGF-β conditioned T-cells and hCD2 (Foxp3)^+^ sorted TGF-β conditioned cells were unable to reject male skin grafts when transferred into lymphopenic B6 Rag1^−/−^ mice (Figure 2C). By contrast, antibody ablation of the hCD2 (Foxp3)^+^ iTreg cells contained within unsorted TGF-β conditioned cultures enabled those remaining cells to reject male grafts rapidly (Figure 2C). Furthermore, sorted hCD2(Foxp3)^−^ TGF-β-conditioned non-Treg cells also rejected rapidly, demonstrating that *in vitro*-induced hCD2(Foxp3)^+^ iTreg cells were competent to suppress all potential pathogenic Foxp3^−^ T cells within the cell mix. To confirm this, we challenged B6 Rag1^−/−^ mice that previously received 10^6^ TGF-β conditioned cells, either unsorted or sorted, with a second CBA Rag1^−/−^ male skin graft and 10^5^ CD4^+^ naive Marilyn T cells. Mice previously treated with *in vitro*-induced Foxp3^+^ iTreg (either sorted or unsorted) maintained long-term graft survival, whereas those receiving hCD2 (Foxp3)^−^ cells rejected their grafts rapidly (Figure 2D). Together, these data demonstrate that *Dby* specific *in vitro-*Foxp3^+^ iTreg fail to reject male skin grafts and are functional suppressors *in vivo*.

It is well recognized that *in vitro*-induced Foxp3^+^ T cells are an unstable population, with the majority of cells losing Foxp3 expression *in vivo* and reacquiring the capacity to generate proinflammatory cytokines (8). The determinants of *in vivo* stability of iTreg are less well understood. Our observation of long-term graft survival and functional suppression *in vivo* suggests that Foxp3 expression in iTreg must be stabilized *in vivo*, at least in some T cells.

**Figure 3:**
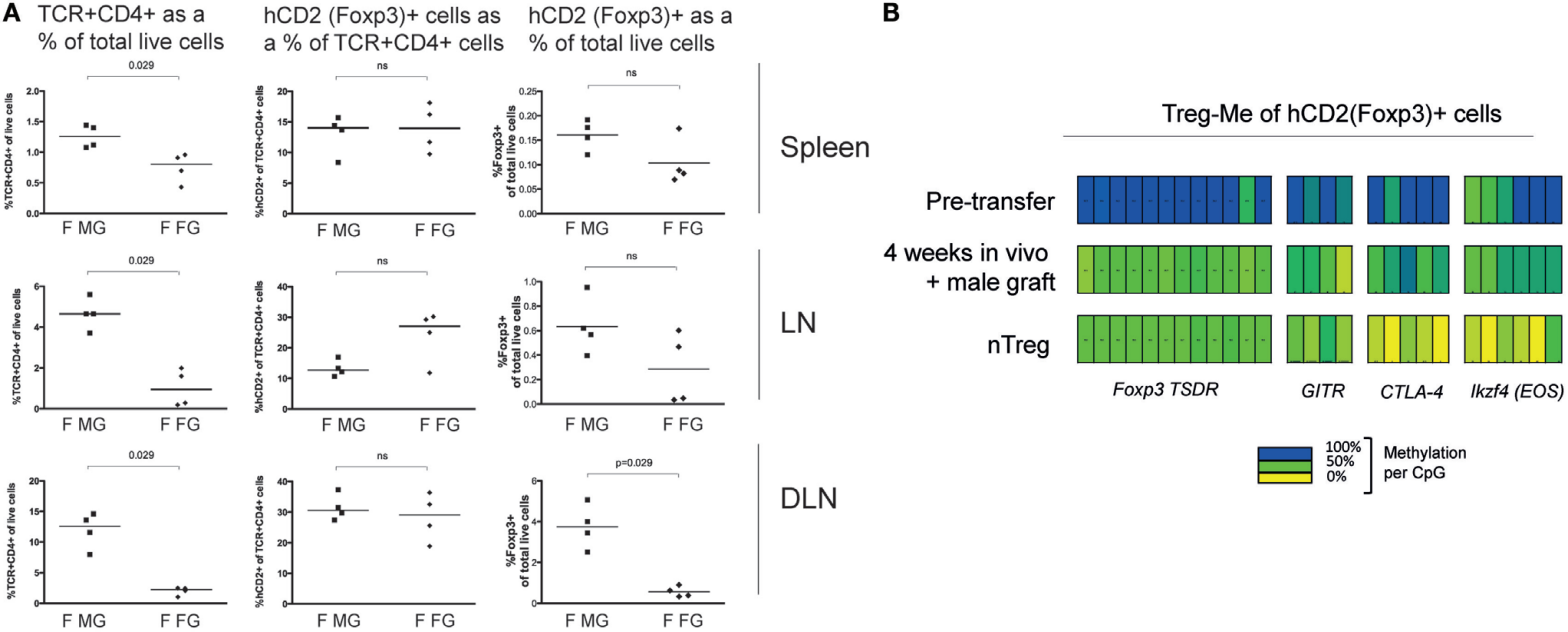
**TGF-β conditioned iTreg acquire Treg-specific hypomethylation status *in vivo***. **(A)** Female B6.RAG1^−/−^ Marilyn.Foxp3^hCD2^ mice received 10^6^ MoFlo sorted Dby-specific iTreg and either a male or female skin graft. Mice were sacrificed at week 4 and spleen, lymph nodes (LN), and draining lymph nodes (DLN) analyzed for the presence of Foxp3^+^ cells. Data are presented as TCR^+^ CD4^+^ cells as a percentage of total live cells, Foxp3^+^ cells as a percentage of TCR^+^ CD4^+^ cells and Foxp3^+^ cells as a percentage of total live cells. “F MG” indicates female recipients with a male graft, “F FG” indicates female recipients with a female graft. Statistical analysis, Mann–Whitney *U* test. **(B)** Treg-specific hypomethylation pattern was assessed in Foxp3^+^ iTreg before transfer and in Foxp3^+^ cells isolated from mice bearing a male skin graft. Results from nTreg are shown as control for adequate comparison. Data representative of three separate experiments.

We investigated the stability of Foxp3 expression *in vivo* and any association with epigenetic changes, as previously observed in nTreg (5). For this purpose, we sorted hCD2^+^ iTreg with >95% purity. When these iTreg were transferred into female B6 Rag1^−/−^, grafted with a male graft, then 4 weeks later, 3.8 ± 0.9% of total live cells in draining lymph nodes maintained Foxp3 expression (Figure 3A). When no antigen was provided, as in the case of a female skin graft, very few (0.6 ± 0.22% of total live cells) still expressed Foxp3 (*p* = 0.029 versus male graft). Similar but non-significant differences were observed in the spleen and non-draining lymph nodes (Figure 3A). This difference was reflected in total TCR^+^ CD4^+^ numbers as a percentage of total live cells. However, the relative percentage of Foxp3^+^ cells within the CD4^+^ T cell pool did not significantly vary between those mice receiving male or female grafts. Together, these data suggest an important role for antigen in enabling the persistence, expansion, and accumulation of CD4 T cells including Foxp3-expressing cells. This is in line with previous observations that the persistent presence of antigen is required to maintain long-term Treg-dependent transplantation tolerance (14, 17).

The instability of iTreg after short-term *in vitro* culture has been attributed to the absence of a required epigenetic signature (5, 8). However, our data suggest that Foxp3 expression can be maintained in some transferred iTreg *in vivo* for an extended period of time and clearly renders some cells able to provide sustained suppression. We investigated the epigenetic “nTreg-Me” signatures in Foxp3^+^ cells isolated from mice which had received iTreg and had not rejected their male grafts. The Foxp3^+^ cell numbers in mice given female skin grafts were simply too few for any epigenetic analysis. Before infusion of the iTreg, there was little evidence of hypomethylation of “nTreg-Me” loci, a finding in line with observations by others (Figure 3B). However, one clone did show full hypomethylation of the TSDR locus suggesting that a small minority of iTreg can acquire an epigenetic hypomethylation pattern even after just 7 days *in vitro* culture (Figure S1 in Supplementary Material). When we assessed the “nTreg-Me” in Foxp3^+^ cells 4 weeks after transfer in mice holding a male skin graft, we observed a hypomethylation pattern that was comparable to that found in wild-type nTreg (Figure 3B). Since cells were derived from female mice, the minimal TSDR methylation expected was around 50%, which was what we actually observed. Collectively, these data demonstrate that some of the T cells-induced *in vitro* to express Foxp3 can be stabilized *in vivo* in circumstances where they are re-exposed to antigen and can acquire epigenetic maturation comparable to wild-type nTreg. The fact that iTreg failed to reject skin grafts and demonstrate functional suppression *in vivo*, even after a challenge with naive CD4^+^ T cells, strongly suggests a sustained commitment to the Treg lineage of at least some of these cells.

Treg-specific hypomethylation has been reported to be ­incomplete in recent thymic emigrants with further epigenetic maturation developing in the periphery (5, 18). The subsequent signals driving progressive epigenetic maturation remain incompletely understood. The development of the epigenetic hallmark of tTreg requires TCR stimulation but is independent of Foxp3 expression (5). Continued TCR signaling is important for maintenance of Treg suppressor function, but not for Foxp3 expression in already established nTreg (19). Furthermore, Ohkura et al. demonstrated (5) that sustained TCR stimulation *in vitro* could also induce partial Treg-specific hypomethylation in some nTreg-Me regions, but not the TSDR in naive CD4^+^ T cells, suggesting that duration rather than intensity of TCR signaling is critical to epigenetic maturation. Our data are not inconsistent with these findings and possibly extend them to iTreg, by demonstrating that more iTreg accumulate in circumstances of renewed antigen exposure than without antigen, and that these show the characteristic hypomethylation pattern of mature Treg. As there were too few Foxp3^+^ T cells available from mice that had received female skin grafts, we were not able to conclude a role for antigen in maintaining the hallmark epigenetic pattern of the iTreg, which had remained.

### Treg-Specific Hypomethylation Can Be Induced by Coreceptor Blockade of Naive T Cells *In Vivo*

**Figure 4:**
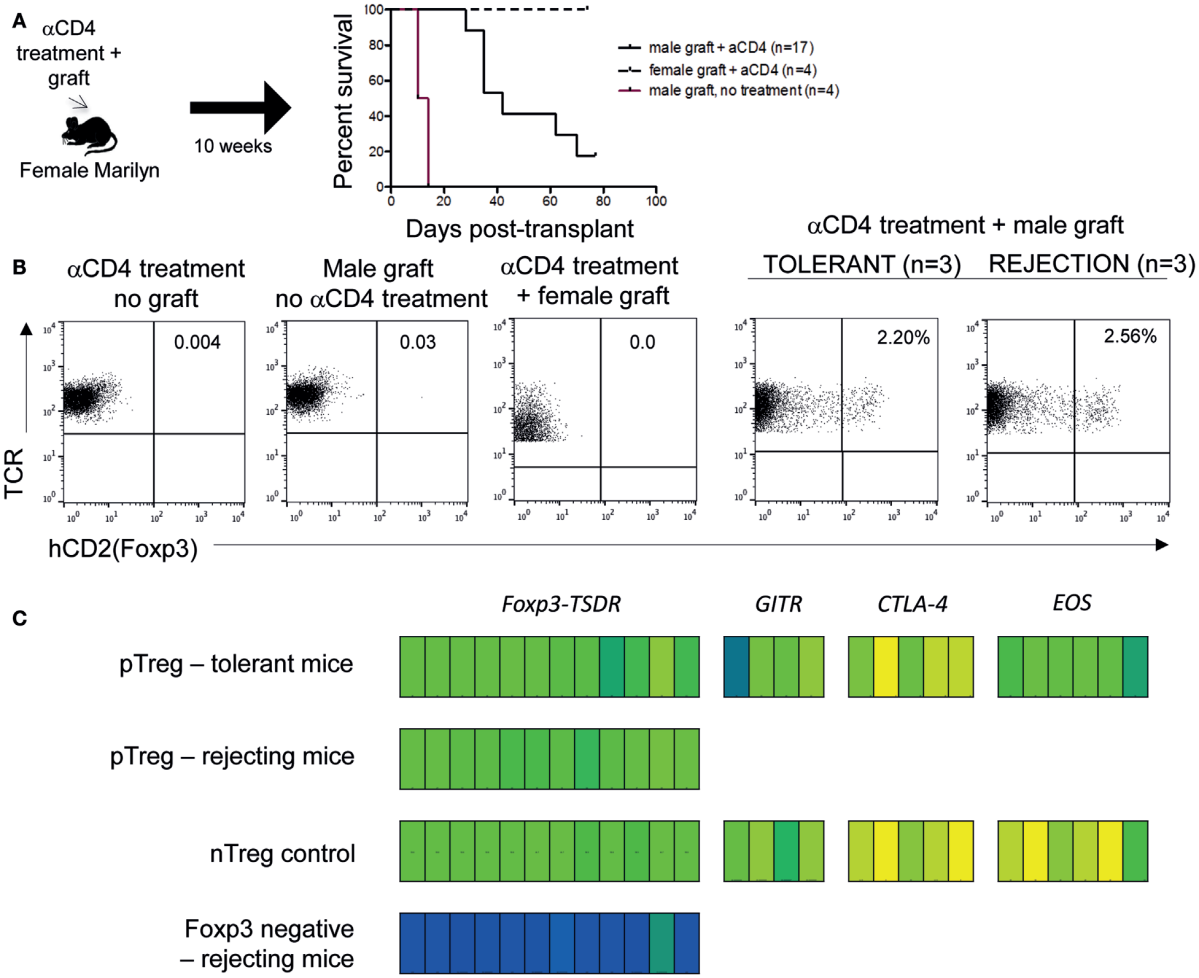
**Antigen exposure in combination with coreceptor blockade generates pTreg with Treg-specific hypomethylation that delay graft rejection**. **(A)** Female B6.RAG1^−/−^ Marilyn.Foxp3^hCD2^ mice were injected intra-peritoneally with 1 mg anti-CD4 (YTS 177.9) at days 0, 3, and 5 and grafted with either male (*n* = 17) or female (*n* = 4) B6.RAG1^−/−^ donor skin. Control mice received male B6.RAG1^−/−^ skin without antibody treatment. Statistical analysis Log-Rank test; *p* < 0.001 for male graft with versus without αCD4 treatment. **(B)** Analysis of Foxp3 expression in draining lymph nodes of treated mice. Data are representative of replicates for mice receiving no male grafts, including two mice receiving anti-CD4 treatment only and four mice receiving female grafts given under cover of anti-CD4. Four mice also received male grafts without anti-CD4 antibody treatment. Plots for anti-CD4 treated mice maintaining (*n* = 3) or rejecting (*n* = 3) male grafts represent pooled data. Similar results were obtained for non-draining lymph nodes and spleen (data not shown). All cells were isolated 10 weeks after grafting. **(C)** Sorted cells were assessed for Treg-specific hypomethylation status of the TSDR locus and “nTreg-Me” loci (iTreg tolerant mice only). nTregs are shown for comparison. Foxp3^−^ cells from rejecting mice demonstrate full TSDR methylation. All cells isolated from mice 10 weeks after grafting.

Adoptive transfer of iTreg has proven effective in enabling transplantation tolerance in rodents (20), but induction of pTreg from within the graft recipient might be more practical for human application. We asked whether pTreg demonstrating an “nTreg-Me” signature could be induced by coreceptor blockade *in vivo* from conventional T cells. To this end, we treated female Marilyn mice receiving a male skin graft with coreceptor blockade (anti-CD4), a treatment that results in long-term graft acceptance *via* the emergence of pTreg (14). In this model, the continuous presence of pTreg is indispensable for long-term graft acceptance, as demonstrated by ablation studies *in vivo* (14). Anti-CD4 treatment resulted in prolonged survival of male skin grafts (Figure 4A) with some treated mice maintaining healthy grafts 10 weeks after treatment. In these mice, Foxp3^+^ cells could be detected in the draining lymph nodes (Figure 4B), non-draining lymph nodes, and spleen (data not shown). Even those mice that eventually rejected male grafts despite antibody treatment showed a similar frequency of Foxp3^+^ cells in secondary lymphoid organs. This could imply that, even despite eventual rejection, there had been a sufficient duration of antigen exposure to stabilize some pTreg. By contrast, Foxp3^+^ cells remained undetectable in mice treated with antibody and a female graft, and, as expected, in mice treated with antibody alone. These data indicate that antigen is needed for *in vivo* induction and does not exclude a role for antigen in the long-term stabilization of Foxp3 expression.

Cells from anti-CD4 treated mice receiving a male skin graft were pooled and analyzed for the hallmark Treg-specific hypomethylation pattern (Figure 4C). Foxp3^+^ cells isolated from mice that tolerated their graft demonstrated an nTreg-Me methylation status comparable to control naive Treg from B6.Foxp3^hCD2^ reporter mice. Of note, anti-CD4 treated mice that rejected their grafts also displayed full demethylation of the TSDR region, suggesting that they too developed pTreg that are epigenetically indistinguishable from nTreg (Figure 4C; Figure S3 in Supplementary Material). Thus, despite the observed induction of pTreg, male grafts, in this experiment, were eventually rejected in a large proportion of mice indicating that there were probably insufficient pTreg to prevent rejection by Foxp3^−^ antigen-specific effector cells. As expected, those T cells that remained Foxp3^−^ after anti-CD4 treatment retained full methylation at the TSDR locus.

Collectively, these data indicate that coreceptor blockade given with the allograft, but not without it, enables induction of pTreg epigenetically similar to nTreg, and by inference, cells that are relatively stable, and exhibit limited plasticity. Our data indicate that this exposure to antigen at the outset, and presumably TCR signaling, is necessary to generate the epigenetically stable pTreg.

## Discussion

In this report, we have shown for the first time that some peripherally induced and *in vitro* generated Treg are stable in the context of renewed exposure to cognate antigen and are epigenetically indistinguishable from nTreg at the Foxp3 TSDR and nTreg-Me. This is the first demonstration of the stability and epigenetic maturation of pure peripheral Treg in an allogeneic transplant setting.

Regulatory T cells expressing Foxp3 require hypomethylation at specific sites to ensure their functional stability over time. This hypomethylation pattern is the most reliable tool that we have today to identify T cells committed to the Foxp3^+^ Treg lineage. Although some of the molecular mechanisms leading toward Foxp3^+^ Treg hypomethylation have been characterized for Treg developing in the thymus, they remain incompletely understood for peripherally-induced Treg, *in vivo*, and have not yet been reported in the setting of transplantation. Functionally, forced demethylation of the TSDR by knocking down DNA methyl transferase 1 (Dnmt1) results in a stable Treg phenotype (5). We studied the acquisition of epigenetic changes both in TGF-β-induced iTreg adoptively transferred into RAG^−/−^ mice and in pTreg developing from naive CD4^+^ T cells in mice lacking any Treg. Our data demonstrate an essential role for antigen in both cell types for Treg maintenance. In the absence of sufficient Foxp3^+^ T-cells accumulating in mice not exposed to the allo-antigen, we cannot rule out any additional role for antigen in maintaining the hallmark epigenetic pattern of Treg.

*In vitro*-induced Foxp3^+^ Treg is a potential source of therapeutic cells for clinical applications but their stability remains uncertain since epigenetic changes are incomplete. Our data demonstrate that *in vitro* TGF-β-induced Foxp3^+^ Treg are unable to reject skin grafts and some cells continue to maintain their epigenetic changes, *in vivo*, resulting in a demethylated TSDR to the same extent as seen in nTreg. Our findings show an important role for the renewed contact with antigen for epigenetically stable cells to accumulate. Previous work has suggested that, in the context of an ongoing autoimmune inflammatory response, autoreactive Treg become unstable over time (21). The inflammatory response to skin engraftment is insufficient in itself to promote pTreg induction or iTreg maintenance as evidenced by lack of pTreg induction and iTreg reduction in numbers in recipients of female grafts. This has precluded us from being able to analyze epigenetic changes in the very rare Foxp3^+^ T-cells that are detectable after such treatments. However, the transient inflammation observed in our model does not inhibit the emergence of pTreg or persistance of iTreg in the presence of cognate antigen. Whether antigen promotes survival or proliferation of Treg cannot be concluded from our study. However, the fact that continuous TCR stimulation is able to induce a certain level of Treg-specific epigenetic changes in naive CD4^+^ T cells *in vitro* over the short term (5) indicates that they might need a longer period of antigen exposure for full maturation or stabilization *in vivo*. These persisting cells may have proliferated more efficiently in the presence of antigen explaining the higher numbers when compared with iTreg levels in mice lacking antigen. Collectively, our data support a role for renewed antigen exposure for sustained suppression by iTreg after *in vivo* transfer, since no rejection occurred despite the presence of effector T cells capable of rejection.

Enhancing Foxp3^+^ Treg numbers within the body is an alternative strategy to promote tolerance that may be more applicable to clinical translation in the long term than parenteral cell infusion-based therapies. Our data demonstrate that *in vivo*-induced antigen-specific Foxp3^+^ Treg can acquire specific hypomethylation, and consequently, lineage commitment and optimal stability. In this model, no Foxp3^+^ tTreg were present at the beginning of the experiment, so any Treg emerging (pTreg) had done so de novo.

It is unknown whether TSDR and nTreg-Me demethylation precede or are consequent to Foxp3 gene transcription during coreceptor blockade induction of pTreg. Treg developing in the thymus have been shown to mature in a two-step fashion with epigenetic maturation starting at early stages of tTreg development that complement Foxp3 expression to ensure stable Treg function. However, both processes occur independently of each other. Moreover, TCR stimulation is clearly required in the early development of nTreg-Me (5) but appears dispensable for the maintenance of the hypomethylation pattern in mature Treg cells (22). These findings suggest that other, yet unidentified factors are required to maintain and maybe also initiate the epigenetic landscape of Treg. Our data support the findings that TCR signaling is essential for the development and accumulation of T-cells with the Treg-specific hypomethylation pattern and confirm that this can be achieved independently of thymic input. It is clear that continuous TCR signaling is required for activated Treg to exert adequate Treg function (19), and the conclusion is also being supported by our study. However, the degree to which TCR signaling is required for ensuring maintenance of the epigenetically mature state remains to be established for tTreg, iTreg, and pTreg. Either way strategies to enhance numbers of pTreg to tame inflammatory diseases may need to take account of a need for recurrent/persistent TCR signaling.

## Ethics Statement

All animal procedures were performed in accordance with the Home Office Animals 10 (Scientific Procedures) Act of 1986 under project license numbers PPL 30/2549 and PPL 11 30/3060. The study was approved by the University of Oxford Animal welfare and ethical review board.

## Author Contributions

RH was involved in experimental design, execution, and writing of the paper. AK, YC, and EA contributed experimental data. HW, SC, and DH were involved in experimental design and writing of the paper.

## Conflict of Interest Statement

The authors declare that the research was conducted in the absence of any commercial or financial relationships that could be construed as a potential conflict of interest.

## Supplementary Material

Figure S1 **Lollipop diagram showing CpG methylation status per clone (8–13 independent clones for each region)**. Female mice demonstrate approximately 50% methylation of the TSDR locus in nTreg by X-chromosome inactivation. Spaces with no “lollipops” represent regions with ambiguous sequence data.

Figure S2 **Lollipop diagram showing CpG methylation status per clone (8–27 independent clones for each region) from iTreg pre-transfer and after retrieval in mice grafted with a male skin graft**. Spaces with no “lollipops” represent regions with ambiguous sequence data.

Figure S3 **Lollipop diagram showing CpG methylation status per clone (9–18 independent clones for each region) from T cells retrieved 10 weeks after treatment with a male skin graft and YTS177.9**. Foxp3^pos^ cells and Foxp3^neg^ cells were analysed from mice where grafts were not rejected at week 10 **(A)** and mice where grafts we rejected **(B)**. Spaces with no “lollipops” represent regions with ambiguous sequence data.
