# Supplementary figures and images for: Induced Foxp3+ T Cells Colonizing Tolerated Allografts Exhibit the Hypomethylation Pattern Typical of Mature Regulatory T Cells
Source: Front Immunol. 2016 Apr 11;7:124. doi: 10.3389/fimmu.2016.00124 (PMC4827454; doi:10.3389/fimmu.2016.00124)

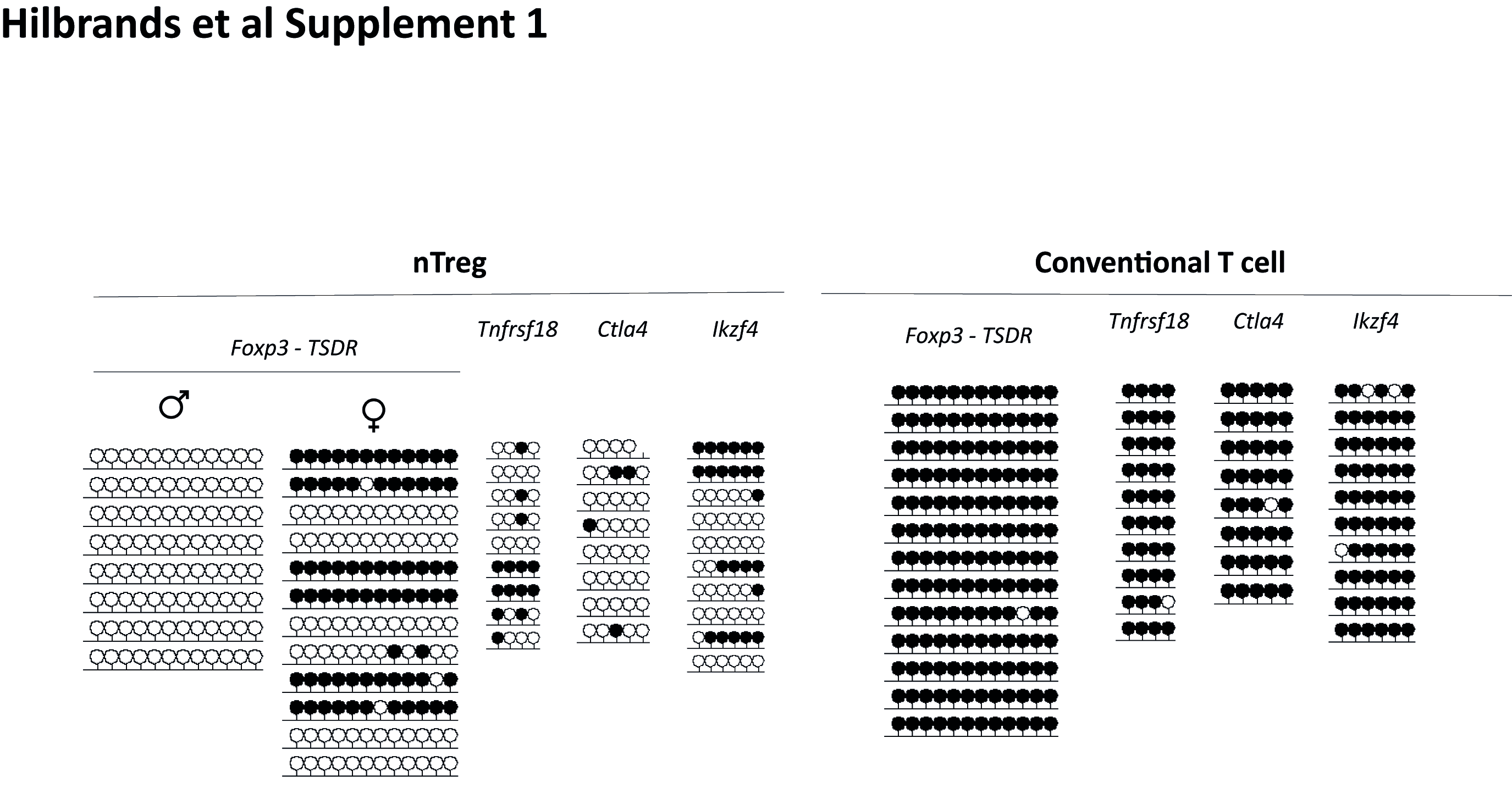

Supplement: Figure S1 — Lollipop diagram showing CpG methylation status per clone (8–13 independent clones for each region). Female mice demonstrate approximately 50% methylation of the TSDR locus in nTreg by X-chromosome inactivation. Spaces with no “lollipops” represent regions with ambiguous sequence data. [file Image_1.TIF]

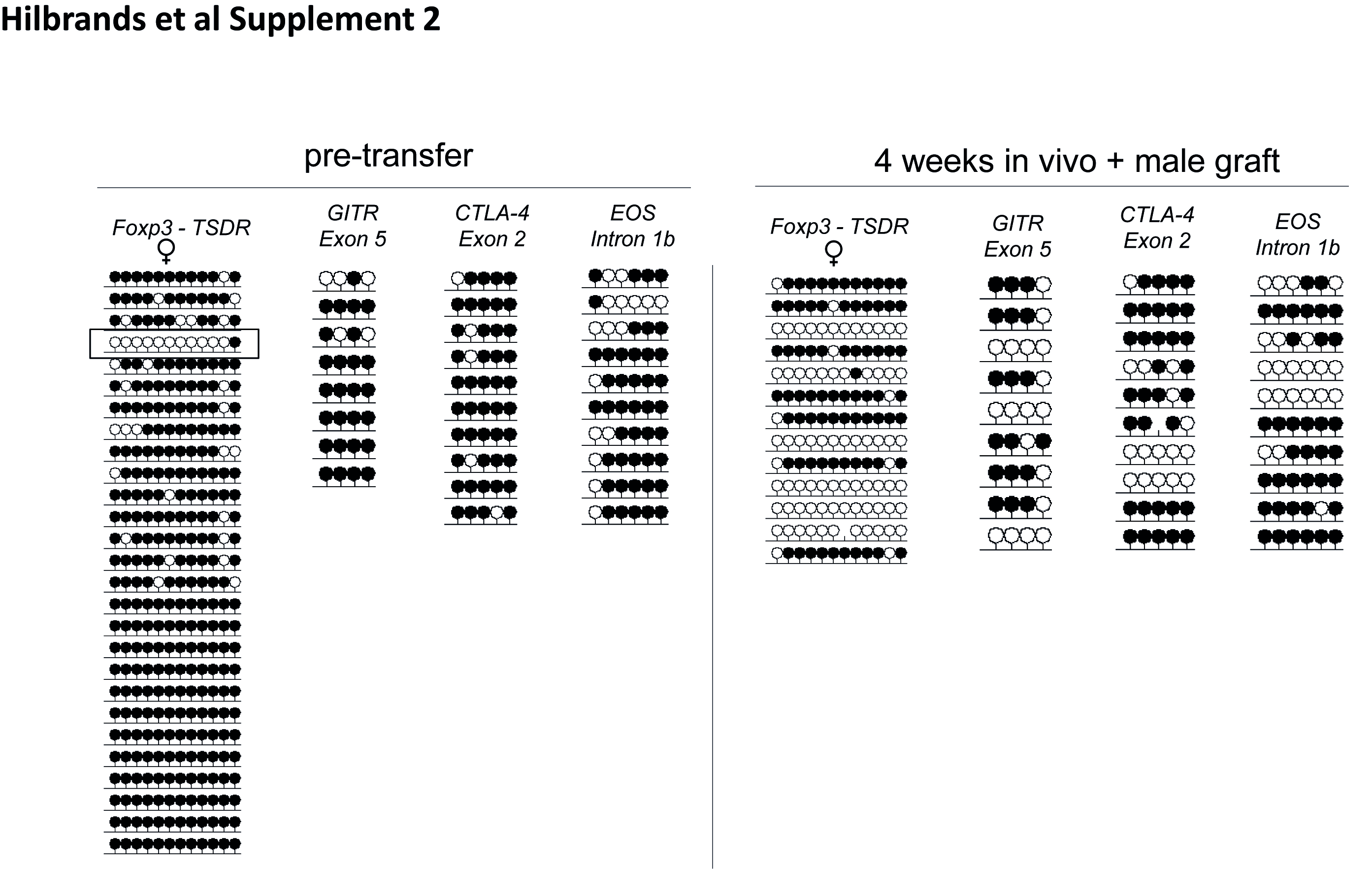

Supplement: Figure S2 — Lollipop diagram showing CpG methylation status per clone (8–27 independent clones for each region) from iTreg pre-transfer and after retrieval in mice grafted with a male skin graft. Spaces with no “lollipops” represent regions with ambiguous sequence data. [file Image_2.TIF]

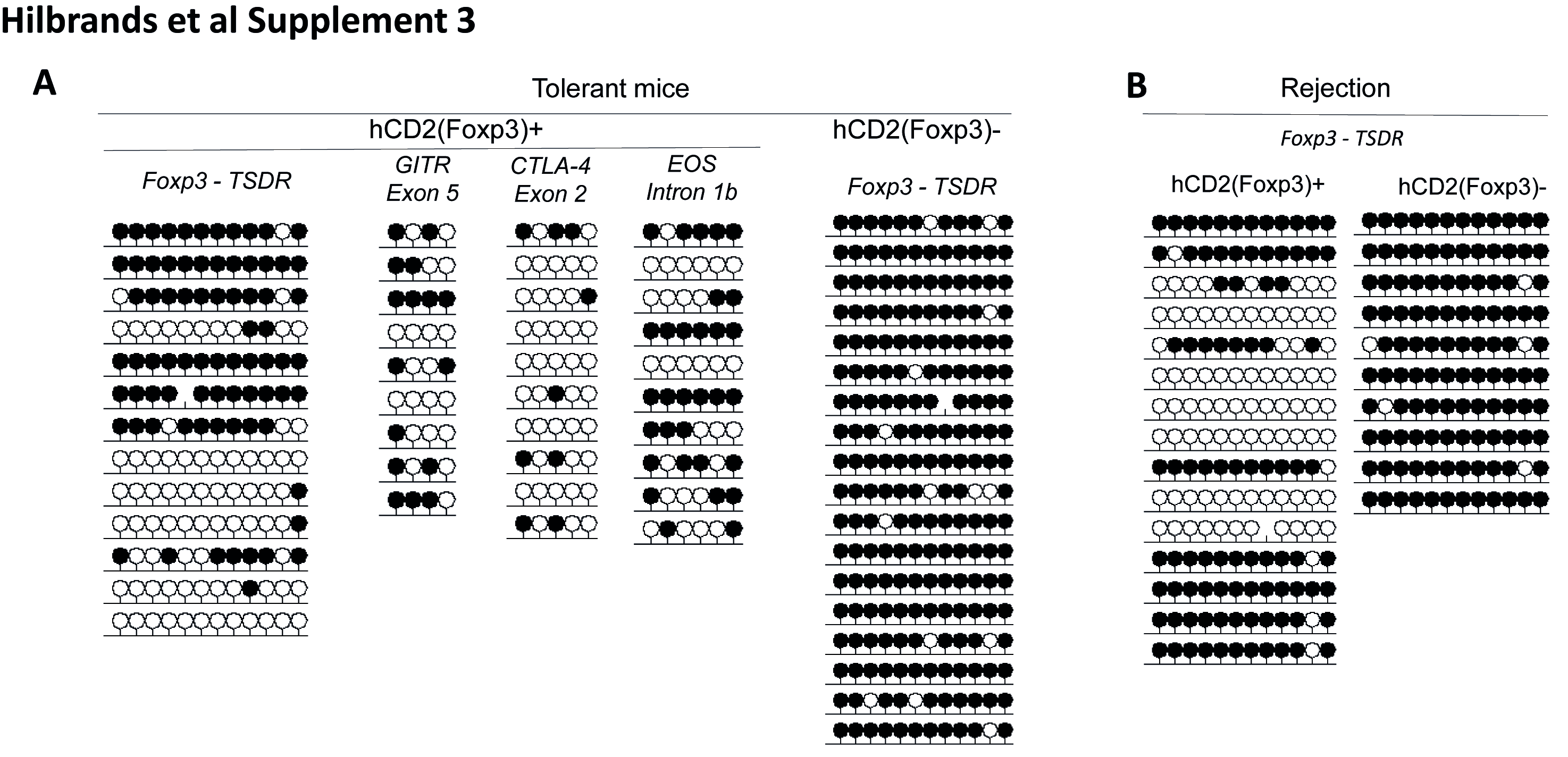

Supplement: Figure S3 — Lollipop diagram showing CpG methylation status per clone (9–18 independent clones for each region) from T cells retrieved 10 weeks after treatment with a male skin graft and YTS177.9. Foxp3pos cells and Foxp3neg cells were analysed from mice where grafts were not rejected at week 10 (A) and mice where grafts we rejected (B). Spaces with no “lollipops” represent regions with ambiguous sequence data. [file Image_3.TIF]
